# The Macrophage A2b Adenosine Receptor Regulates Tissue Insulin Sensitivity

**DOI:** 10.1371/journal.pone.0098775

**Published:** 2014-06-03

**Authors:** Hillary Johnston-Cox, Anna S. Eisenstein, Milka Koupenova, Shannon Carroll, Katya Ravid

**Affiliations:** 1 Department of Medicine, Boston University School of Medicine, Boston, Massachusetts, United States of America; 2 Department of Biochemistry, Boston University School of Medicine, Boston, Massachusetts, United States of America; 3 Whitaker Cardiovascular Institute, Boston University School of Medicine, Boston, Massachusetts, United States of America; 4 Evans Center for Interdisciplinary Biomedical Research, Boston University School of Medicine, Boston, Massachusetts, United States of America; Warren Alpert Medical School of Brown University, United States of America

## Abstract

High fat diet (HFD)-induced type 2 diabetes continues to be an epidemic with significant risk for various pathologies. Previously, we identified the A2b adenosine receptor (A2bAR), an established regulator of inflammation, as a regulator of HFD-induced insulin resistance. In particular, HFD was associated with vast upregulation of liver A2bAR in control mice, and while mice lacking this receptor showed augmented liver inflammation and tissue insulin resistance. As the A2bAR is expressed in different tissues, here, we provide the first lead to cellular mechanism by demonstrating that the receptor's influence on tissue insulin sensitivity is mediated via its expression in macrophages. This was shown using a newly generated transgenic mouse model expressing the A2bAR gene in the macrophage lineage on an otherwise A2bAR null background. Reinstatement of macrophage A2bAR expression in A2bAR null mice fed HFD restored insulin tolerance and tissue insulin signaling to the level of control mice. The molecular mechanism for this effect involves A2bAR-mediated changes in cyclic adenosine monophosphate in macrophages, reducing the expression and release of inflammatory cytokines, which downregulate insulin receptor-2. Thus, our results illustrate that macrophage A2bAR signaling is needed and sufficient for relaying the protective effect of the A2bAR against HFD-induced tissue inflammation and insulin resistance in mice.

## Introduction

The prevalence of type 2 diabetes mellitus has risen rapidly in the United States in parallel to the obesity epidemic [1], [2]. There is an increased risk of cardiovascular disease in type 2 diabetics [3] and in patients with metabolic syndrome [4]. Recent studies have explored the role of macrophages in inflammatory processes, such as atherosclerosis and insulin resistance [5], [6]. Chronic inflammation that is associated with obesity has been shown in mice and humans to contribute to the pathogenesis of type 2 diabetes [7]–[10]. Cytokines, like tumor necrosis factor-alpha (TNF-α) and interleukin-6 (IL-6), which are secreted by pro-inflammatory macrophages in the adipose tissue and liver of obese individuals, increase the inhibitory serine phosphorylation of insulin receptor substrate (IRS)-1 and -2 and reduce levels of IRS-2 [11]–[13]. This results in reduced insulin signaling and subsequent insulin resistance [11], [13]–[21]. The importance of obesity-induced inflammation in the development of type 2 diabetes is underscored by studies that show that inhibition of macrophage activation or infiltration into adipose tissue and liver ameliorates insulin resistance, while an increase in macrophage infiltration or activity aggravates insulin resistance [7]–[9], [22]–[28].

We have previously created [29] and characterized mice that lack the A2b adenosine receptor (A2bAR) in the context of high fat diet (HFD)-induced insulin resistance [30]. This G-protein coupled receptor activates adenylyl cyclase to increase cyclic adenosine monophosphate (cAMP) levels upon adenosine binding. A2bAR knockout (KO) mice have an increased inflammatory profile at baseline and after lipopolysaccharide (LPS) injection [29]. Moreover, we showed that activation of macrophage A2bAR reduces the expression of TNF-α [31]. We recently published that after HFD, A2bAR KO mice have reduced glucose clearance, elevated plasma glucose, peripheral tissue insulin resistance, and elevated inflammatory cytokines [30]. In search of a mechanism for this effect, we found that A2bAR KO mice have decreased levels of IRS-2 in adipose tissue and liver [30]. Cytokines released from macrophages, such as TNF-α, are known to reduce levels of IRS-2. Therefore, in the current study, we tested the hypothesis that the contribution of A2bAR signaling in macrophages is paramount in conveying the protective effect of the A2bAR in HFD-induced insulin resistance and glucose homeostasis. To test this contention we generated and analyzed a transgenic mouse model in which the human A2bAR gene is expressed in the monocyte lineage using the CD68 promoter [32]–[34] on an A2bAR KO background (hereafter referred to as CD68-Tg) to assess whether expression of A2bAR in macrophages alone would ameliorate the impaired insulin signaling in A2bAR KO mice. Our studies show that macrophage A2bAR signaling plays a significant role in the protective effect of A2bAR in HFD-induced insulin resistance.

## Materials and Methods

### Mice

All mice procedures were carried out in strict accordance with the recommendations in the Guide for the Care and Use of Laboratory Animals of the National Institutes of Health. The protocol (#AN-14064) was approved and in agreement with the guidelines of the Institutional Animal Care and Use Committee of the Boston University School of Medicine. In all studies, age-matched male mice were used. For metabolic experiments, 12-week-old mice were subjected to HFD (42% kcal fat, 42.7% kcal carbohydrates, 15.7% kcal protein, supplemented with 0.2% cholesterol; Teklad, cat# TD88137) for 16 weeks. The A2bAR KO/β-galactosidase knock-in mouse model used in these studies has been generated by our laboratory as previously described [29]. Matching wild type (WT) (also on C57Bl/6J background) mice were bred in our facility, originating initially from cross breeding A2bAR KO mice with WT mice on the same genetic background. The WT controls were not from the same litter as the A2bAR KO mice, but were age-, sex- and strain-matched.

Transgenic mice were generated to express human A2bAR in macrophages on an A2bAR KO background. The CD68-A2bAR-β-Globin polyA construct was designed and constructed as follows: SM22α gene promoter was excised using NotI and HindIII from a SM22α-hA2BAR-β-Globin polyA construct. The gene promoter, CD68, was excised from a CD68-intron-A2aAR construct in a pcDNA vector (gift obtained from Dr. Chen, BUSM) using BsrGI and ClaI. The promoter in the vector was gene-cleaned (Q.Biogene, 1102-999-3), blunted with the Klenow fragment of DNA polymerase I (NE Biolabs, #Mo210S), and 5′ ends were dephosphorylated with 1 unit Antartic Phosphatase (NE Biolabs, #M0289S) per 1 µg of vector DNA. The vector and promoter were ligated overnight at 16 °C in a 1∶3 molar ratio with T4 ligase (NE Biolabs, #M0202S). DH5α competent cells (Invitrogen, #18265-017) were transformed with 100 ng of vector. Colonies were selected and were grown overnight. The bacterial DNA was isolated with QIAprep Spin Miniprep Kit. Insertion and orientation of the CD68 promoter was verified by enzyme digestion and DNA sequencing confirmed the final product.

### Isolation of Tissues

Mice were starved for 16 hours, anesthetized, and organs were collected for sectioning (fixed in 4% paraformaldehyde) or snap-frozen for further analyses. For peripheral tissue insulin signaling (phosphorylation of Akt), mice were injected with 1 U/kg Humulin R (Lilly, U-100, #0002-8215-91) 15 minutes before collection. In the case of adipose tissue isolation for tissue sectioning, mice were transcardially perfused with 10 mL of PBS, followed by 20 mL of 4% paraformaldehyde.

### Liver Kupffer Cells and Adipose Tissue Macrophages

One liver lobe was excised from WT, A2bAR KO, and CD68-Tg mice. The liver lobes were minced and digested in RPMI 1640 media containing 5% FCS and 0.03% weight/vol, collagenase type IV (Sigma Aldrich, cat. #C5138) at 37 °C for 1 hour. Resulting extract was passed through 100 µm mesh and placed on ice for 10 minutes. The following procedures were carried out at 4 °C. Cell suspension was spun at 200 x g for 5 minutes. The supernatant was removed and spun at 800 x g for 10 minutes. The supernatant was aspirated and the pellet resuspended in 10 mL of RPMI 1640. Kupffer cells were recovered by a 25%/50% Percoll gradient and spun at 800 x g for 15 minutes. Cell suspension from the gradient was plated in RPMI 1640, 1% penicillin-streptomycin (Fisher, cat. #30-001-CT) (1×10^6^ cells per 24-well plate), incubated for 30 minutes at 37 °C and then media was changed to remove non-adherent cells. Primary Kupffer cells were pretreated with adenosine deaminase (1 U/mL final concentration, Roche Applied Science, #10102105001) and papaverine hydrochloride (5 mM, Sigma, #P3510) for 10 min, and subsequently treated with the indicated pharmacological compound. After 10 min of treatment, cells were collected and cAMP was measured (Direct cAMP EIA Kit, Assay Design). cAMP levels were normalized to total protein by Bradford protein assay (Bio-Rad, cat# 500-0006). As to adipose tissue macrophages, the epididymal adipose tissue was extracted from mice after 16 weeks of HFD for isolation of adipose tissue macrophages in the stromal vascular fraction [35]. The adipose tissue was minced in type IA collagenase (1 mg/mL in 1X HBSS) and then digested for 1 hour at 37 °C, with frequent agitation. The digested tissue was then passed through a 100 µm filter and centrifuged at 500 x g for 10 min at 4 °C. The supernatant and floating lipid layer was aspirated. The pellet was resuspended in 2 mL red blood cell lysis buffer and incubated at 37 °C for 10 minutes, followed by centrifugation for 5 min at 500 x g at 4 °C. The resulting pellet was then resuspended in MACS buffer (0.5% BSA, 2 mM EDTA) at a concentration of 10×10^6^ cells/mL. Cell staining for flow cytometry was performed as in [35]. Briefly, cells were blocked with anti-mouse CD32/CD16 Fcγ (1∶100; eBioscience 14-0161-81) for 15 min at 4 °C. Cells were labeled with CD11c-PE (1∶300; eBioscience cat# 12-0114-81), CD206-Alexa 647 (1∶300; AbD Serotec cat# McA2235A647T) and F4/80-FITC (1∶300; eBioscience cat# 11-4801-81) for 30 min at 4 °C. Cells were sorted on a BD FACSAria II SORP cell sorter. Three populations were collected: (1) F4/80+, CD11c+, CD206-; (2) F4/80+, CD11c-, CD206+; (3) F4/80+, CD11c+, CD206+. The sorted cells were pelleted and frozen at −80 °C prior to RT-PCR analysis.

### Metabolic Analyses

Prior to glucose tolerance test (GTT), mice were starved for 16 hours. Tail blood glucose was measured with a Glucometer (One Touch Ultra) before and at 15, 30, 45, 60, 90, 120 minutes post intraperitoneal injection of glucose (1.25 g/kg). Insulin clearance was measured in the plasma (cheek bleed) before and at 20 and 120 minutes post glucose injection. Insulin levels were measured in 5 µl of plasma using ALPCO Insulin EIA kit (cat # 80-INSMSU-E01). Prior to insulin tolerance test (ITT), mice were starved for 6 hours. As described above, tail blood glucose was measured before and at 15, 30, 60, 90, 120 minutes post intraperitoneal injection of insulin (1 U/kg, Humulin R U-100, Lilly).

### Body-composition analysis

Body composition was assessed using nuclear magnetic resonance (NMR) measurements (EchoMRI, Echo Medical Systems, Houston, TX, USA), and EchoMRI software (version 2007.08.10) at baseline (12 weeks of age) and following 16 weeks of HFD. The instrument was calibrated prior to each measurement session, using a special phantom provided by the manufacturer. NMR measurements were made by placing live mice into a thin wall plastic cylinder (4.7 cm, inside diameter; 0.15 cm thick), with freedom to turn about but limited to ∼4-cm vertical movements by a plastic insert. Relative lean mass and fat mass values were calculated by dividing absolute values by body weight.

### Plasma TNF-α Levels

Mice were anesthetized by isoflurane, blood collected by cardiac puncture and plasma isolated [36]. Plasma TNF-α was measured according to manufacturer's instructions (eBioscience, cat #88-7324).

### Quantitative Polymerase Chain Reaction (qPCR)

Tissue RNA was extracted as follows: a small piece of tissue was homogenized in 350 µL of buffer RLT Plus supplemented with β-mercaptoethanol or Qiazol for adipose tissue. RNA was isolated from the homogenate with RNeasy Plus Mini kit (Qiagen cat# 74134) or RNeasy Lipid Tissue Mini kit (Qiagen cat# 74804) and cDNA was generated using the High Capacity cDNA Reverse Transcription Kit (Life Technologies cat# 4368813) or QuantiTect Reverse Transcription kit (Qiagen cat #205311), according to manufacturer's instructions. Messenger RNA levels of genes were quantified using Applied Biosystems (AB) TaqMan primers. The following Taqman primers were used: mouse A2bAR (mA2bAR, Mm00839292_m1); human A2bAR (hA2bAR, Hs00386497_m1); mouse TNF-α (Mm99999068_m1); mouse IL-6 (Mm00446191_m1); mouse monocyte chemotactic protein 1 (MCP1, Mm00441242_m1); mouse CD68 (Mm03047340_m1); mouse IRS-2 (Mm03038438_m1). Levels of the expression for each gene were normalized to 18s rRNA (AB part #4319413E) with the TaqMan Gene Expression Master Mix (Life Technologies cat# 4370048), using the ABI 7300 Real-Time PCR System. The amplification efficiency of mA2bAR and hA2bAR primers was determined using the C_t_ slope method. mA2bAR and hA2bAR Taqman primers were found to have equal efficiencies.

### Western Blots

Pieces of liver lobe and adipose tissue (∼0.5 cm^3^) were homogenized (using Power Gen 1000, #10-008781, speed 2, 15 seconds) with 300 µl of RIPA buffer (1× PBS, 1% NP-40, 0.5% sodium deoxycholate, 0.1% SDS, 10 mg/ml PMSF, and aprotinin [2 µg/ml]) supplemented with protease and phosphatase inhibitors (0.5 mM NaVa_3_, 100 nM Okadaic acid, 50 mM NaF, 5 mM Na-pyro-P0_4_). The lysate was left on ice for 30 min, vortexed for 5 min and centrifuged at 16,000 x g for 10 min. The supernatant was assayed for protein content by Bradford assay reagent. An aliquot of the supernatant (typically, 100 µg protein) was mixed with an equal volume of 2X sodium dodecyl sulfate (SDS) loading buffer and resolved by 8%-SDS PAGE for total IRS-2 protein and 10%-SDS PAGE for all other proteins. Membranes were incubated overnight with primary antibody at a dilution of 1∶1000: anti-rabbit SREBP-1 (Novus Biologicals, cat# NB100-2215), anti-rabbit IRS-2 (Cell signaling, cat#3089S), anti-rabbit pAkt (Ser473) (Cell signaling, cat# 4060P), anti-rabbit pAkt (Thr308) (Cell signaling, cat# 2965P), and anti-rabbit total Akt (Cell Signaling, cat# 4691P). Membranes were washed and incubated for an hour with the appropriate secondary antibodies from Santa Cruz: goat-anti-rabbit (cat# sc2004); goat-anti-mouse (cat# sc2060); donkey-anti-goat (cat# sc2020). Proteins were visualized with chemiluminescent reagent kit (Millipore, cat# WBKLS0500). Blots were stripped, re-blocked and re-blotted with 1∶10,000 dilution anti-mouse β-actin (Sigma, cat# A5441) to confirm equal loading.

### Plasma and Liver analysis for cholesterol, triglycerides and glycerol

Cholesterol, triglycerides, and glycerol were measured as previously described [36].

### Tissue Histology

For F4/80 immunostaining, paraffin sections of visceral fat post HFD were fixed in 10% paraformaldehyde and embedded as described in [29]. Sections were deparaffinized and then analyzed with F4/80 macrophage antibody as described previously [29]. Briefly, heat induced antigen retrieval was performed with 10 mM Citric Acid (pH 6.0). Sections were blocked with Avidin/Biotin solution (Vector Laboratories kit, #SP-2001) and 10% goat serum (Vector Laboratories kit, #SP-2001). The sections were incubated with primary antibody F4/80 (1∶500, AbD Serotec, #mcA497R) overnight at 4°C, followed by biotinylated anti-goat secondary antibody (1∶200, Vector Laboratories, #BA-9400) for 30 minutes, ABC-AP reagent (Vector Laboratories kit, #AK-5001) for 30 minutes, and vector red alkaline phosphate substrate (Vector Laboratories kit, #AK-5001) for 15 minutes and then counterstained with hematoxylin and eosin (H&E). Liver tissue was similarly fixed and embedded, and then sections were stained with H&E.

### Statistical Analysis

The data from each experiment is expressed as means ± standard deviation (SD). Statistical comparison was done using two-tailed Student t-test. The results were considered significant when the means were different with p<0.05 (*). When appropriate and as indicated, we used one-tailed ANOVA followed by the Bonferroni multiple-comparison test. Statistical analyses were performed with Excel or GraphPad Prism5 software.

## Results

### Generation of a Mouse Model Expressing A2bAR in Macrophages

**Figure 1:**
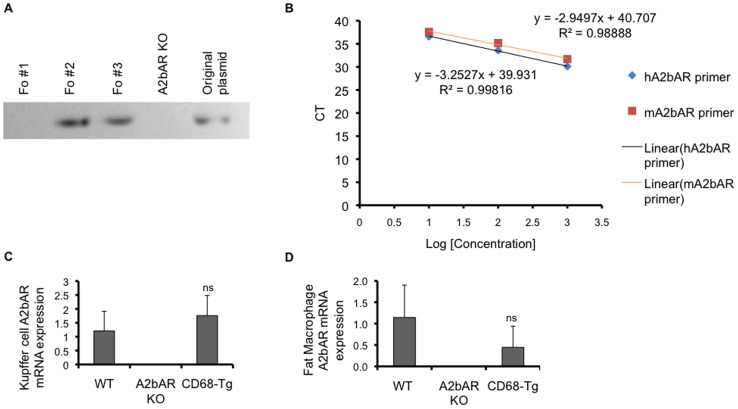
Generation of transgenic mice expressing A2bAR in macrophages only. **A**. Genomic analysis by PCR of CD68-hA2bAR transgene in founder lines 1, 2, and 3 (Fo # 1, 2, 3) compared to A2bAR KO mice. Line 2 was used for the remainder of the studies based on expression analysis shown in panels c,d. **B**. Determination of primer efficiency. The amplification efficiency of human A2bAR (hA2bAR) and mouse A2bAR (mA2bAR) TaqMan primers was tested using the CT slope method. The target template was diluted over a log scale and CT values were determined by qPCR. A plot of CT versus log cDNA concentration is shown for hA2bAR and mA2bAR primers. Amplification efficiency (Ex) is calculated using the slope of the graph in the following equation: Ex = 10 (-1/slope) – 1. The calculated efficiencies are 1.18 and 1.03 for mA2bAR and hA2bAR, respectively. **C**. Human A2bAR and mouse A2bAR mRNA expression was measured by qPCR in Kupffer cells isolated from mice at 12 weeks of age (n = 5 WT, 6 A2bAR KO, 6 CD68-Tg; ns = not statistically different from WT). **D**. Visceral (epididymal) adipose tissue macrophages were sorted via flow cytometry-based markers (see Methods) and subjected to qPCR of A2bAR mRNA. Data are averages ± SD. Relative mRNA expression was determined using the ΔΔCT method and were normalized to 18s rRNA values. Data are averages ± SD. *Student two-tail t-test assuming equal variance was found significant only when p-value <0.05.

Kupffer cells and adipose tissue macrophages have been shown to play a role in obesity-induced insulin resistance in the liver and fat, respectively [8], [16], [22]–[26]. As we had previously reported a protective role for A2bAR in maintaining metabolic homeostasis in mice on HFD [30], and considering the expression of this receptor in macrophages, we sought to explore the specific role of the A2bAR in macrophages in mediating this protective effect. To examine whether gain of A2bAR function in macrophages restores the protective effect of the A2bAR, we generated transgenic mice (CD68-Tg) that express A2bAR only in the monocyte lineage by using the CD68 gene promoter to drive human A2bAR expression on an A2bAR KO background. The CD68 promoter has been used in the past to direct expression of a transgene to macrophages [32], [37], [38]. The goal was also to specifically explore the role of human A2bAR, by “humanizing” a mouse model. A founder line showing macrophage A2bAR activation similar to WT was used for this study (Figure 1A). We focused on analysis of tissue macrophages, considering their contribution to tissue insulin sensitivity. Given the similar efficiencies of the human and mouse Taqman primers (Figure 1B), we compared the expression of A2bAR in Kupffer cells, resident liver macrophages. A2bAR expression was similar in Kupffer cells from WT and CD68-Tg mice (Figure 1C). Further, visceral adipose tissue macrophages sorted by flow cytometry (considering their scarcity) showed similar expression of A2bAR in the CD68-Tg and WT mice (Figure 1D).

### Analysis of CD68-Tg Tissue Macrophages Demonstrates Increased cAMP Activity and Reduced Inflammation

**Figure 2:**
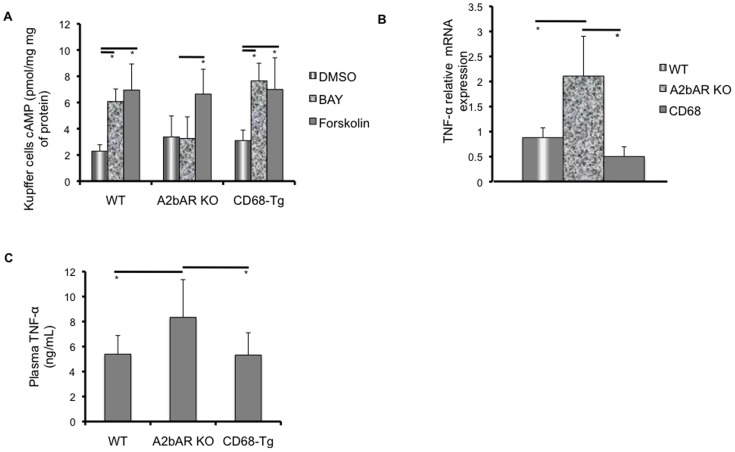
Effect of macrophage A2bAR on TNF-α. Kupffer cells were extracted from the liver of WT, A2bAR KO, and CD68-Tg mice following 16 weeks of HFD. **A**. cAMP levels (normalized to total protein) were assessed as described in the methods following treatment of Kupffer cells with the following ligands for 10 minutes: Dimethylsulfoxide (DMSO), 1 µM BAY 60–6583 (BAY), and 2 µM Forskolin (FSK). n = 5 per genotype per treatment. WT: BAY relative to DMSO p = 0.001738, FSK relative to DMSO p-value  = 0.000932; A2bAR KO: FSK relative to DMSO p-value  = 0.026068; CD68-Tg: BAY relative to DMSO p-value  = 0.000387, FSK relative to DMSO p-value  = 0.010989. **B**. Expression of TNF-α in Kupffer cells. CD68-Tg vs A2bAR KO p-value  = 0.0164, WT vs A2bAR KO p-value  = 0.0032. **C**. Systemic inflammatory profile was assessed in WT, A2bAR KO, and CD68-Tg mice after 16 weeks of HFD. Plasma TNF-α levels were lower in WT, p-value  = 0.0269, and CD68-Tg, p-value  = 0.0291, as compared to A2bAR KO after HFD. Data are averages ± SD. *Student two-tail t-test assuming equal variance was found significant only when p-value <0.05.

Signaling through the A2bAR has been shown to reduce macrophage secretion of cytokines [31], [39]. Furthermore, the downstream effecter of A2bAR signaling, cAMP, is known to reduce macrophage cytokine expression [40]–[43]. Therefore, we determined whether reinstating A2bAR in macrophages resulted in changes in cAMP levels and subsequent cytokine release. The A2bAR specific agonist, BAY 60–6583, was able to elicit an increase in cAMP levels in Kupffer cells from WT and CD68-Tg mice (Figure 2A). Moreover, Kupffer cells that express A2bAR (from WT and CD68-Tg mice) showed reduced mRNA expression of TNF-α as compared to Kupffer cells that lack A2bAR (Figure 2B). Restoration of macrophage A2bAR in CD68-Tg mice was also able to reduce systemic plasma TNF-α (Figure 2C). Hence, our results suggest that expression of A2bAR in Kupffer cells augments cAMP signaling and reduces macrophage cytokine expression and secretion.

### Reduced Tissue Inflammation Alters IRS-2 and SREBP-1 Levels

We next asked if restoration of macrophage A2bAR altered the levels of proinflammatory cytokines at the tissue level. Proinflammatory cytokines, such as TNF-α and IL-6 inhibit insulin signaling, in large part, through their action on the level and activity of IRS-1/2 in hepatocytes and adipocytes [11]–[13]. In addition, TNF-α has been shown to regulate the transcription of SREBP-1, and has also been shown to increase SREBP-1 level [44]–[46]. Therefore, we also sought to determine if tissue levels of IRS-2 and SREBP-1 were subsequently altered.

**Figure 3:**
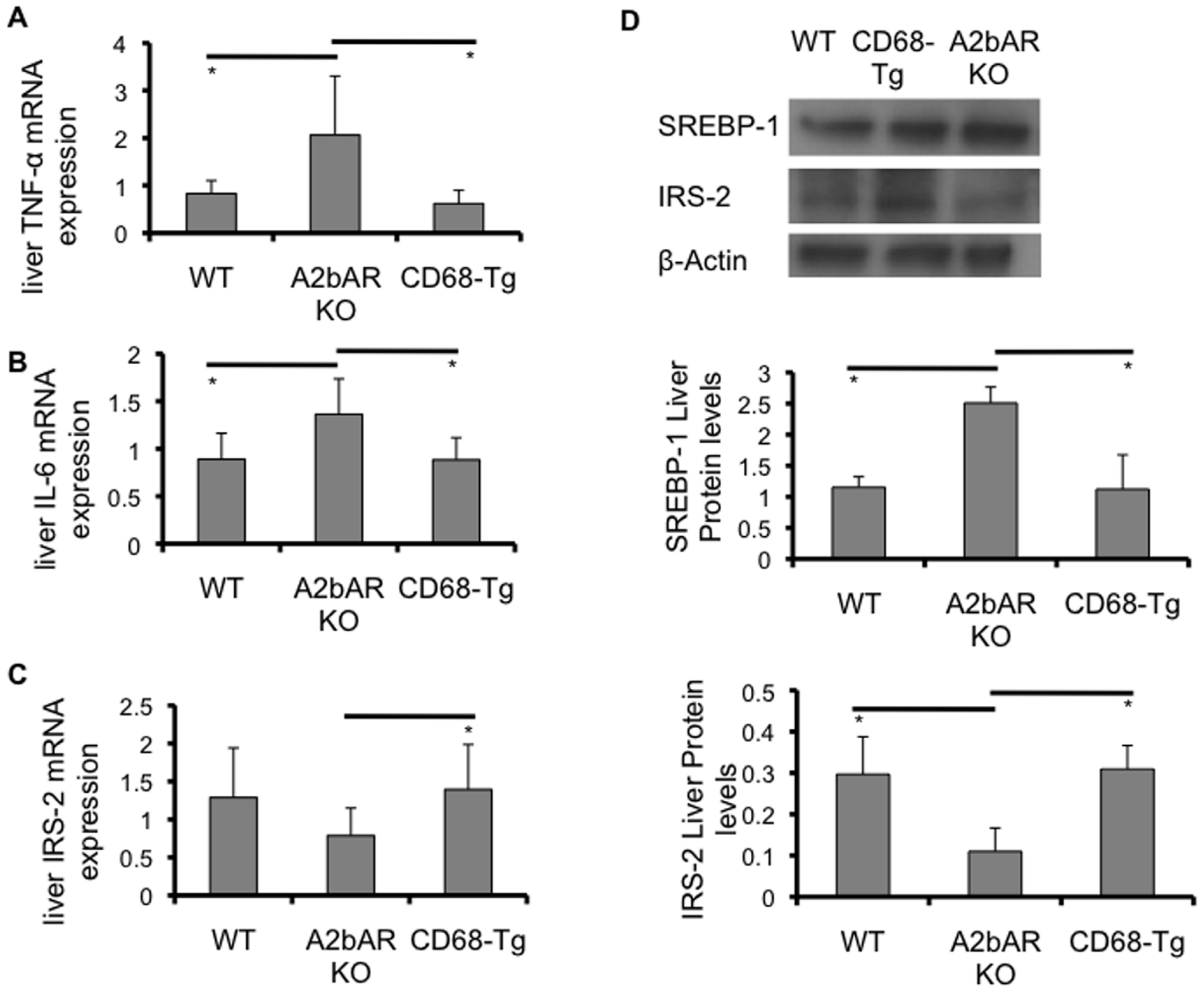
Effect of restoration of macrophage A2bAR on liver. Liver was collected from WT, A2bAR KO, and CD68-Tg mice after 16 weeks of HFD as described in the methods. Relative mRNA expression was determined using the ΔΔCT method with normalization to 18s rRNA. **A**. mRNA expression of TNF-α in liver. A2bAR KO (n = 5) vs WT (n = 7) p-value  = 0.0271; A2bAR KO vs CD68-Tg (n = 6) p-value  = 0.0210. **B**. mRNA expression of IL-6 in liver. A2bAR KO (n = 8) vs WT (n = 7) p-value  = 0.0159; A2bAR KO vs CD68-Tg (n = 8) p-value  = 0.0080. **C**. mRNA expression of IRS-2 in liver. CD68-Tg (n = 7) vs A2bAR KO (n = 8) p-value  = 0.0300. **D**. Western blot analysis of liver; one representative (of 3 sets) WT, CD68-Tg and A2bAR KO group shown at 15 minutes post-insulin injection, following 16 weeks of HFD. Levels of mature SREBP-1 (68 kDa), and IRS-2 (185 kDa), were probed by Western blot analysis, using β-actin (43 kDa) as loading control. Quantification of Western Blot results was performed with Image J software (http://rsb.info.nih.gov/ij/) with normalization to β-actin. WT to A2bAR KO: IRS-2 p-value  = 0.0092, SREBP-1 p-value  = 0.0154; CD68-Tg to A2bAR KO: IRS-2 p-value  = 0.0247, SREBP-1 p-value  = 0.0170. Data are averages ± SD. *Student two-tail t-test assuming equal variance was found significant only when p-value <0.05.

**Figure 4:**
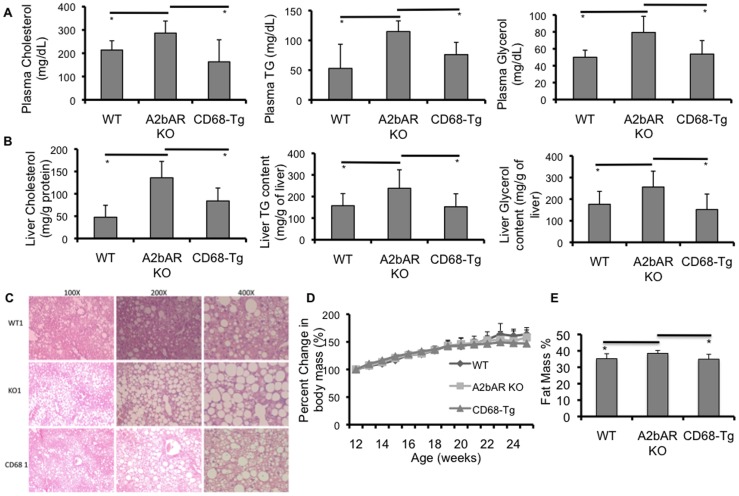
Macrophage A2bAR affects plasma and hepatic lipids and percent fat mass. Plasma and liver were collected from WT (n = 6), A2bAR KO (n = 6), and CD68-Tg (n = 6) male mice following 16 weeks of HFD. Plasma and liver cholesterol and triglyceride (TG) was measured as described in the methods. H&E staining of paraffin embedded liver was performed as in the methods and body mass was determined weekly Data are averages ± SD. *Student two-tail t-test assuming equal variance was found significant only when p<0.05. **A**. Plasma cholesterol, TG and glycerol levels. Cholesterol: CD68-Tg vs A2bAR KO p-value  = 0.0351, WT vs A2bAR KO p-value  = 0.0463. TG: CD68-Tg vs A2bAR KO p-value  = 0.0446, WT vs A2bAR KO p-value  = 0.0313. Glycerol: CD68-Tg vs A2bAR KO p-value  = 0.0218, WT vs A2bAR KO p-value  = 0.0074. **B**. Liver cholesterol, TG and glycerol content. Cholesterol: CD68-Tg vs A2bAR KO p-value  = 0.0463, WT vs A2bAR KO p-value  = 0.0125. TG: CD68-Tg mice vs A2bAR KO p-value  = 0.0291, WT vs A2bAR KO p-value  = 0.0352. Glycerol: CD68-Tg mice vs A2bAR KO p-value  = 0.0158, WT vs A2bAR KO p-value  = 0.0317. **C**. Liver morphology at a magnification of 100, 200, and 400x. **D**. Percent increase in body mass on HFD did not differ between genotypes. **E**. Percent fat mass were measured by NMR for WT, A2bAR KO, and CD68 transgenic mice, N = 8 per group. There is an increase in percent fat mass p-value  = 0.0393 in A2bAR KO mice as compared to WT mice and as compared to CD68-Tg mice p-value  = 0.0171. Data are averages ± SD. *Student two-tail t-test assuming equal variance was found significant only when p-value <0.05.

Interestingly, expression of TNF-α and IL-6 in the liver of CD68-Tg mice was reduced significantly and mildly, respectively, compared to A2bAR KO mice (Figure 3A,B). In accordance, Western blot and qPCR analyses of the liver showed increased IRS-2 protein and mRNA levels in CD68-Tg mice as compared to A2bAR KO mice, mimicking the levels found in WT mice (Figure 3C,D). We also found reduced liver SREBP-1 protein expression in the CD68-Tg mice as compared to A2bAR KO mice (Figure 3D), which corresponds to the diminished cytokine expression in the CD68-Tg mice. SREBP-1 plays a crucial role in lipid metabolism. To this end, restoration of macrophage A2bAR reduced plasma levels and liver content of triglyceride and cholesterol in CD68-Tg as compared to A2bAR KO mice (Figure 4A,B). Moreover, lipid accumulation in the livers of A2bAR KO mice was ameliorated with restoration of macrophage A2bAR (Figure 4C) despite no change in weight gain on HFD between genotypes (Figure 4D). Consistent with resolution of hyperlipidemia and reduction in liver triglyceride and cholesterol content, WT and CD68-Tg mice demonstrated lower percent fat mass relative to A2bAR KO mice (Figure 4E).

**Figure 5:**
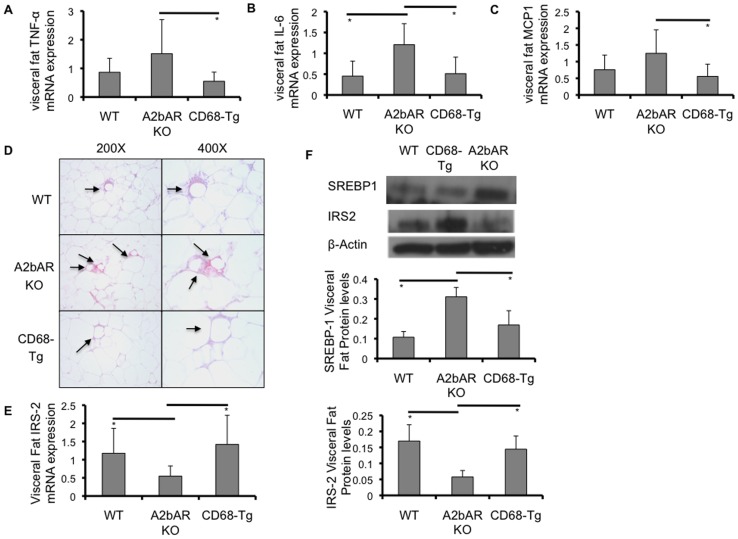
Effect of restoration of macrophage A2bAR on adipose tissue. Visceral (epididymal) adipose tissue was collected from WT, A2bAR KO, and CD68-Tg mice after 16 weeks of HFD as described in the methods. **A-C,E.** Relative mRNA expression was determined using the ΔΔCT method with normalization to 18s rRNA. **A**. mRNA expression of TNF-α in visceral fat. A2bAR KO (n = 7) vs CD68-Tg (n = 9) p-value  = 0.0345. **B**. mRNA expression of IL-6 in visceral fat. A2bAR KO (n = 7) vs WT (n = 7) p-value  = 0.0073; A2bAR KO vs CD68-Tg (n = 7) p-value  = 0.0141. **C**. mRNA expression of MCP1 in visceral fat. A2bAR KO (n = 7) vs CD68-Tg (n = 9) p-value  = 0.0232. **D**. Visceral adipose tissue from WT, A2bAR KO, and CD68-Tg mice after 16 weeks of HFD was fixed in 4% paraformaldehyde and paraffin-embedded. Sections immunostained with the macrophage marker F4/80. Representative sections for each genotype at a magnification of 200x and 400x. Arrows point to crown-like structures. **E**. mRNA expression of IRS-2 in visceral fat. CD68-Tg (n = 8) vs A2bAR KO (n = 8) p-value  = 0.0115, WT (n = 6) vs A2bAR KO p-value  = 0.0358. **F**. Western blot analysis of visceral fat; one representative (of 3 sets) WT, CD68-Tg and A2bAR KO group shown at 15 minutes post-insulin injection, following 16 weeks of HFD. WT to A2bAR KO: IRS-2 p-value  = 0.0411, SREBP-1 p-value  = 0.0103. CD68-Tg to A2bAR KO: IRS-2 p-value  = 0.0305, SREBP-1 p-value  = 0.0459. Data are averages ± SD. *Student two-tail t-test assuming equal variance was found significant only when p-value <0.05.

As adipose tissue macrophages have been reported to influence glucose homeostasis [47]–[49], we expected that restoration of A2bAR in macrophages might also affect fat tissue metabolic function. Much like in the liver, we found reduced TNF-α and IL-6 mRNA levels in the adipose tissue from CD68-Tg mice as compared to A2bAR KO mice (Figure 5A,B). In addition, as compared to adipose tissue from A2bAR KO mice, that from CD68-Tg mice showed lower expression of MCP1 (Figure 5C), a macrophage chemokine, as well as reduced crown-like structures (Figure 5D). As in the liver, adipose tissue IRS-2 and SREBP-1 levels were restored in CD68-Tg mice to that of WT mice (Figure 5E,F).

### Improved Metabolic Phenotype Following HFD in CD68-Tg Mice

**Figure 6:**
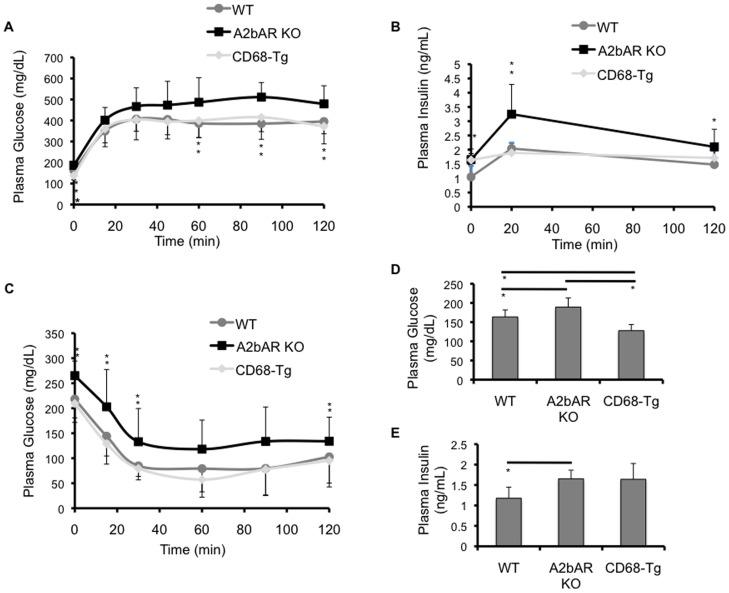
Restoration of macrophage A2bAR improves glucose clearance, insulin sensitivity and reduces glucose levels following HFD. Glucose and insulin levels were measured in WT, A2bAR KO, and CD68-Tg mice at 28 weeks of age, following 16 weeks of HFD. Data are averages ± SD. *Student two-tail t-test assuming equal variance was found significant only when p-value <0.05. A. Glucose clearance in the blood post glucose overload (n = 12/group), Data in a. were analyzed by ANOVA followed by Bonferroni comparison test with the following p-values for indicated time points, Time 0 min: p-value <0.0001; WT vs CD68-Tg p-value <0.05, WT vs A2bAR KO p-value <0.05, CD68-Tg vs A2bAR KO p-value <0.01. Time 60 min: p-value 0.003487; WT vs CD68-Tg n.s., WT vs A2bAR KO p-value <0.01, CD68-Tg vs A2bAR KO p-value <0.01. Time 90 min: p-value 0.005068; WT vs CD68-Tg n.s., WT vs A2bAR KO p-value <0.01, CD68-Tg vs A2bAR KO p-value <0.05. Time 90 min: p-value 0.005855; WT vs CD68-Tg n.s., WT vs A2bAR KO p-value <0.05, CD68-Tg vs A2bAR KO p-value <0.05. B. Insulin levels in the plasma post glucose overload during the glucose tolerance test (n = 12/group). Data in b. were analyzed by ANOVA followed by Bonferroni comparison test with the following p-values for indicated time points. Time 0 min: p-value 0.017963; WT vs CD68-Tg p-value <0.05, WT vs A2bAR KO p-value <0.05, CD68-Tg vs A2bAR KO n.s. Time 20 min: p-value 0.017304; WT vs CD68-Tg n.s., WT vs A2bAR KO p-value <0.05, CD68-Tg vs A2bAR KO p-value <0.05. Time 120 min: p-value 0.019056; WT vs CD68-Tg n.s., WT vs A2bAR KO p-value <0.05, CD68-Tg vs A2bAR KO n.s. C. Glucose clearance in the plasma post insulin overload (n = 12/group). Data in c. were analyzed by ANOVA followed by Bonferroni comparison test with the following p-values for indicated time points. Time 0 min: p-value 0.006653; WT vs CD68-Tg n.s., WT vs A2bAR KO p-value <0.05, CD68-Tg vs A2bAR KO p-value <0.01. Time 15 min: p-value 0.017975; WT vs CD68-Tg n.s., WT vs A2bAR KO p-value <0.05, CD68-Tg vs A2bAR KO p-value <0.05. Time 30 min: p-value 0.016828; WT vs CD68-Tg n.s., WT vs A2bAR KO p-value <0.05, CD68-Tg vs A2bAR KO p-value <0.05. Time 120 min: p-value 0.003444; WT vs CD68-Tg n.s., WT vs A2bAR KO p-value <0.05, CD68-Tg vs A2bAR KO p-value <0.01. D. Plasma glucose levels 16 hours post starvation (n = 12/group). WT vs A2bAR KO, p-value  = 0.0404; WT vs CD68-Tg, p-value  = 0.0074; CD68-Tg vs A2bAR KO, p-value  = 0.0026. E. Plasma insulin levels 16 hours post starvation (n = 12/group), WT vs A2bAR KO p-value  = 0.0175.

**Figure 7:**
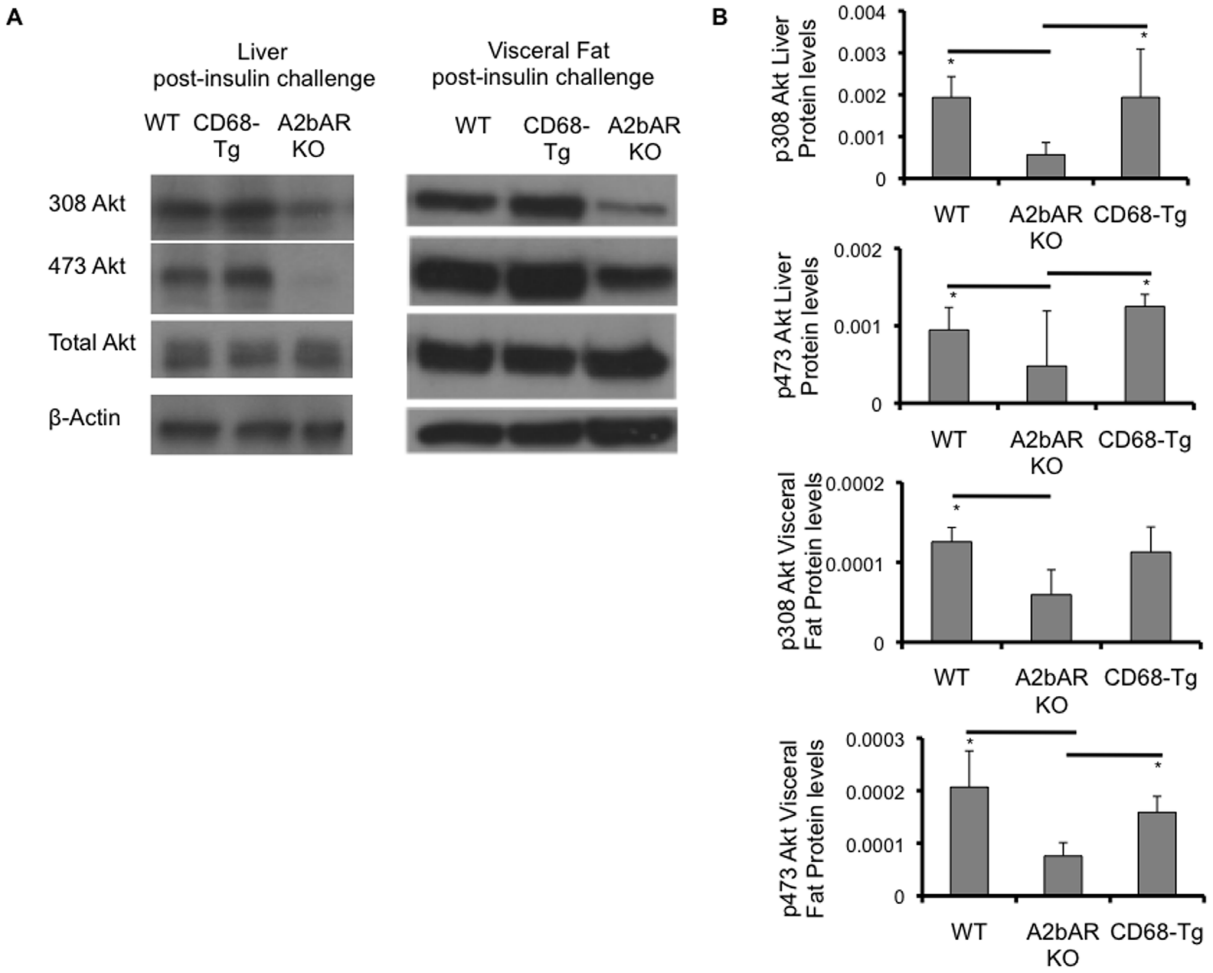
Effect of CD68-driven expression of A2bAR on tissue insulin signaling. Western blot analysis of liver and visceral fat derived from matching WT, CD68-Tg and A2bAR KO mice post 16 weeks of HFD and 15 minutes following insulin injection. **A**. Levels of phospho-308 Akt (p308 Akt, 60 kDa), phospho-473 Akt (p473 Akt, 60 kDa), and total Akt (60 kDa) were probed by Western blot analysis, using β-actin (43 kDa) as loading control. Shown are representative out of 3 sets. **B**. Quantification of Western Blot results was performed with Image J software (http://rsb.info.nih.gov/ij/). Protein levels were normalized to total Akt and β-actin. Data are averages ± SD. *Student two-tail t-test assuming equal variance was found significant only when p<0.05. WT to A2bAR KO: Liver p308 Akt p-value  = 0.0007, p473 Akt p-value  = 0.0219; Visceral fat: p308 Akt p-value  = 0.0340, p473 Akt p-value  = 0.0395; CD68-Tg to A2bAR KO: Liver p308 Akt p-value  = 0.0327, p473 Akt p-value  = 0.0349; Visceral fat: p308 Akt p-value  = 0.1054; p473 Akt p-value  = 0.0221.

As we had found reduced inflammation and increased IRS-2 levels in the CD68-Tg mice, we next determined whether restoration of macrophage A2bAR affected global metabolic homeostasis. Following HFD feeding, CD68-Tg mice showed improved glucose clearance and insulin sensitivity relative to A2bAR KO mice and responded to insulin and glucose no differently than WT mice (Figure 6A-C). Fasting glucose levels were lower in the CD68-Tg mice as compared to A2bAR KO mice, whereas there was no difference in fasting insulin levels between CD68-Tg and A2bAR KO mice (Figure 6D-E). In fact, CD68-Tg mice had lower fasting glucose levels than WT mice. To determine if tissues were insulin resistant, Akt phosphorylation in liver and adipose tissue, which is indicative of insulin signaling [50], was measured. Western blot analysis of liver and visceral adipose tissue after HFD and following injection with insulin demonstrated that tissue insulin signaling was restored to that of WT mice in CD68-Tg mice as the levels of phosphorylated Akt 308 and 473 were similar in CD68-Tg and WT mice (Figure 7A,B). Thus, macrophage A2bAR expression was largely responsible for the protective effect of A2bAR signaling in HFD-induced insulin resistance and glucose tolerance.

**Figure 8:**
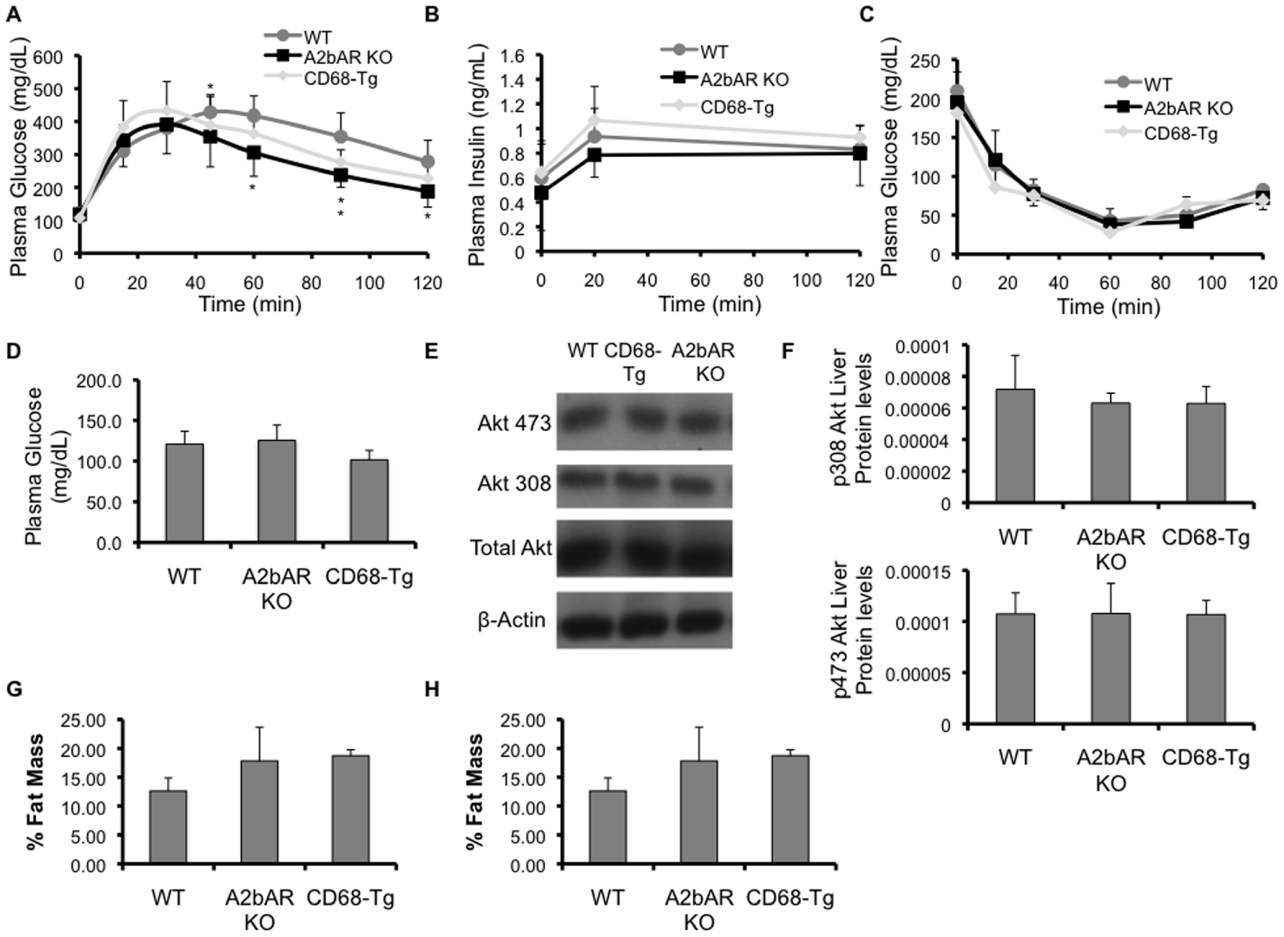
Baseline characteristics of CD68-Tg mice. Baseline characteristics were measured in WT, A2bAR KO, and CD68-Tg mice at 12 weeks of age. **A**. Glucose clearance in the blood post glucose overload (n = 12/group). Data in a. were analyzed by ANOVA followed by Bonferroni comparison test with the following p-values for indicated time points: Time 45 min: p-value  = 0.100922; WT vs CD68-Tg n.s., WT vs A2bAR KO p-value <0.05, CD68-Tg vs A2bAR KO n.s.; Time 60 min: p-value  = 0.003404; WT vs CD68-Tg n.s., WT vs A2bAR KO p-value <0.01, CD68-Tg vs A2bAR KO n.s.; Time 90 min: p-value  = 0.002867; WT vs CD68-Tg p-value <0.05, WT vs A2bAR KO p-value <0.01, CD68-Tg vs A2bAR KO n.s.; Time 120 min: p-value  = 0.016218; WT vs CD68-Tg n.s., WT vs A2bAR KO p-value <0.05, CD68-Tg vs A2bAR KO n.s. **B**. Insulin levels in the plasma post glucose overload (n = 12/group). **C**. Glucose clearance in the plasma post insulin overload (n = 12/group), post 6 hours starvation. **D**. Glucose levels post 16 hour starvation. **E**. Levels of liver phospho-308 Akt (p308 Akt, 60 kDa), phospho-473 Akt (p473 Akt, 60 kDa), and total Akt (60 kDa) were probed by Western blot analysis, using β-actin (43 kDa) as loading control. Shown are representatives out of 3 sets. **F**. Quantification of Western Blot results (of panel E) was performed with Image J software (http://rsb.info.nih.gov/ij/). Protein levels were normalized to total Akt and β-actin. **G**. Fat to Lean ratio at 12 weeks of age. **H**. Percent fat mass relative to body weight at 12 weeks of age. Data are averages ± SD. *Student two-tail t-test assuming equal variance was found significant only when p-value <0.05. The differences between the experimental groups (CD68-Tg or A2bAR KO vs. WT, or CD68-Tg vs A2bAR KO) were not statistically significant.

We also determined the response of CD68-Tg mice to glucose and insulin overload in chow-fed 12-week old mice. There was no difference in glucose clearance (Figure 8A), glucose-stimulated insulin release (Figure 8B), insulin sensitivity (Figure 8C), fasting glucose level (Figure 8D) or liver insulin signaling (assessed by Akt phosphorylation; Figure 8E,F) between the CD68-Tg and A2bAR KO mice. In addition, there was no difference in fat to lean ratio or percent lean mass between CD68-Tg and A2bAR KO mice (Figure 8G,H). These results were not unexpected as the expression of the A2bAR is quite low or not detectable in tissues such as liver or fat under chow diet, while it is upregulated following conditions that stress the animal, such as ischemia, inflammation, and HFD [29], [30], [51]-[53]. Notably, and as previously described [54], glucose clearance under chow diet is more efficient in the A2bAR KO mice compared to control WT mice. This could be due to an inhibitory effect of low level A2bAR in chow diet-fed mice on tissue IL-6 expression, as suggested in [54].

**Figure 9:**
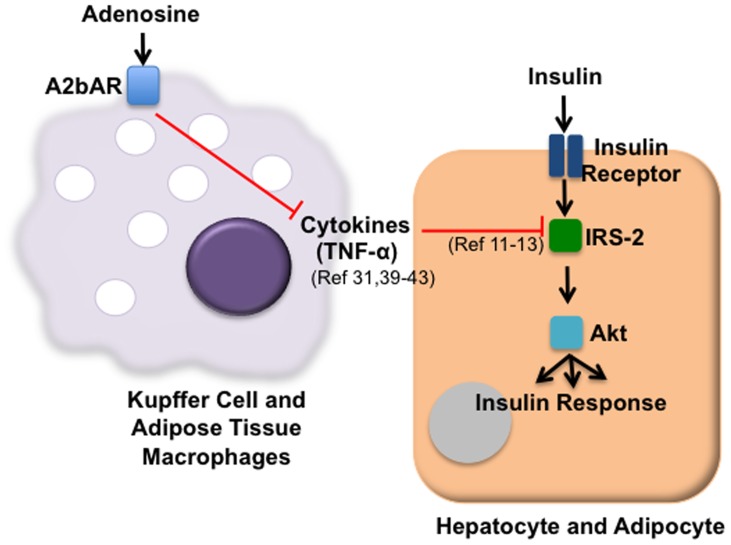
Proposed mechanism for preventative action of A2bAR on insulin signaling. Macrophage signaling through the A2bAR reduces TNF-α expression [31], [39]–[43]. Reduced cytokine expression prevents the inflammation-induced decrease in tissue IRS-2 by the direct action of inflammatory cytokines [11]–[13]. As a result, macrophage A2bAR signaling enables appropriate tissue insulin signaling.

Taken together, our findings suggest that the monocytic lineage is the major cellular player in conveying A2bAR-induced protection against insulin resistance. Our findings suggest that under HFD, signaling through macrophage A2bAR reduces inflammatory cytokine expression via increased cAMP levels, which leads to augmented levels of IRS-2 in adipocytes and hepatocytes, ultimately leading to improved insulin signaling as outlined in Figure 9.

## Discussion

Macrophages have long been implicated in controlling glucose and insulin homeostasis (reviewed in [21]). Several recent studies have highlighted how control of macrophage signaling, via c-Jun N-terminal kinase (JNK) and Notch ligand Delta-like 4 (DLL4), affects inflammation and insulin resistance [55], [56]. Purinergic signaling has previously been shown to play a role in regulating inflammation and metabolic disease. As compared to control, mice lacking CD39, the main ectoenzyme that converts ATP and ADP to AMP (which is then further metabolized to adenosine), had more severe glucose intolerance and insulin resistance that was associated with increased inflammation [57]. This effect may be due to an increase in ATP or as is consistent with our findings, a decrease in adenosine levels. The current study illustrates the ability of macrophage A2bAR to regulate glucose and insulin homeostasis, and protect against the development of insulin resistance.

Total body knockout of the A2bAR results in worsened insulin sensitivity and glucose tolerance in mice fed a HFD [30]. We now establish that restoration of A2bAR expression on macrophages alone is sufficient to restore the response to glucose and insulin challenge to levels of WT mice. There is contention within the literature regarding the expression of CD68 in lineages other than the monocyte/macrophage lineage [58], [59]. The use of the humanized CD68 promoter to drive gene expression in macrophages has been utilized by previous studies, demonstrating significant expression in macrophages and specific to the monocyte/macrophage lineage [32], [33], [60]. Given these studies and the established high expression of the A2bAR in macrophages [29], [31], the CD68 promoter was used to drive macrophage A2bAR expression with the studies predominantly focusing on this lineage. We have previously shown that A2bAR KO mice are pro-inflammatory [29] and inflammation has been shown to play an important role in the development of insulin resistance and type 2 diabetes [13], [17], therefore, we hypothesized that reduction in macrophage-induced inflammation in the A2bAR KO mice would improve metabolic parameters in HFD-fed mice. Consistent with this hypothesis, CD68-Tg mice had decreased tissue inflammation in association with improved glucose and lipid homeostasis as compared to A2bAR KO mice. Percent fat mass was restored in the CD68-Tg mice relative to WT consistent with improved lipid profile. Our lab has also shown that activation of macrophage A2bAR reduces TNF-α secretion *in vitro* and *in vivo* [31]. Given our previous and current findings, we propose that activation of the A2bAR decreases macrophage cytokine (such as TNF-α) release from macrophages in liver and adipose tissue. This prevents cytokine-induced reduction in levels of IRS-2 [11], [13], [61] in the hepatocytes and adipocytes. When IRS-2 levels are maintained, insulin signaling and whole body response to glucose and insulin challenge improves (Figure 9). In the CD68-Tg mice, reinstating macrophage A2bAR reduced tissue inflammation, increased liver and adipose tissue IRS-2 levels, and restored tissue insulin signaling to that of control mice.

In addition to improvement in insulin sensitivity and glucose disposal, restoration of macrophage A2bAR also reduced serum glucose levels post-starvation. In fact, plasma glucose levels in CD68-Tg mice were significantly less than that found in WT mice. This result can be explained by improved tissue insulin signaling in the face of elevated insulin secretion, a finding observed in the total A2bAR KO mice [30]. Macrophage A2bAR appears to be protective for tissue insulin sensitivity and glucose disposal, while A2bAR signaling in different cell types (e.g. pancreatic beta cells) is pivotal for other aspects of glucose homeostasis, such as insulin secretion. The A2bAR has been recently demonstrated to be integral for regulation of adipokines and classical and alternative macrophage activation, demonstrating that impaired glucose and lipid metabolism in A2bAR KO mice involved increased classical activation of macrophages [62]. This underscores the concept that the role of the A2bAR in the pathogenesis of insulin resistance and glucose homeostasis is multifaceted. Previous studies investigating a role for A2bAR in metabolic homeostasis have not elucidated adipocyte or hepatocyte specific mechanisms in regards to inflammation and metabolic disease [36], [54], [62]. Thus, our study sought to determine the cell type responsible for mediating the protective role of the A2bAR in metabolic responses in the liver and adipose tissue. Of note, we performed fluorescence-activated cell sorting experiments of adipose tissue macrophages derived from the above genotypes and did not see differences in percent of F4/80+, CD11c+ (M1) or F4/80+, CD206+ (M2) macrophages (pro- and anti-inflammatory), and thus concluded that differential macrophage distribution in fat is not responsible for the phenotype in A2bAR KO mice (data not shown).

In summary, in this and other studies [30], [36] we have demonstrated several protective aspects of the A2bAR against metabolic disease and cardiovascular disease following HFD. Our findings underscore the importance of A2bAR signaling in macrophages in modulating glucose and lipid homeostasis and suggest that manipulation of macrophage A2bAR, either pharmacologically or by genetic manipulations of monocytes, may be a promising therapeutic approach.
